# Orthotopic Liver Transplantation in a Patient With *GALT* p.Ser135Leu/Null

**DOI:** 10.1002/jmd2.70016

**Published:** 2025-05-09

**Authors:** Kara Simpson, Erin L. MacLeod, Julia Clayton, Nada A. Yazigi, M. Estela Rubio‐Gozalbo, Judith L. Fridovich‐Keil, Gerard T. Berry, Nicholas Ah Mew

**Affiliations:** ^1^ Division of Genetics and Metabolism Children's National Hospital Washington DC USA; ^2^ Medstar Georgetown Transplant Institute Georgetown University Hospital Washington DC USA; ^3^ MosaKids Children's Hospital, Department of Pediatrics, Laboratory of Clinical Genetics Maastricht University Medical Center Maastricht the Netherlands; ^4^ Department of Human Genetics Emory University School of Medicine Atlanta Georgia USA; ^5^ Division of Genetics and Genomics Boston Children's Hospital Boston Massachusetts USA

**Keywords:** galactose‐1‐phosphate, galactose‐1‐phosphate uridylyltransferase deficiency, galactosemia, liver transplant, newborn screening

## Abstract

We report the case of a now 12‐year‐old male compound heterozygous for a novel *GALT* null variant and the p.Ser135Leu variant, associated with clinical variant galactosemia. This patient presented with fulminant liver failure at age 2 months requiring liver transplant. Despite initial detection by newborn screening, a misinterpretation of results led to delayed diagnosis and treatment. While the p.Ser135Leu *GALT* variant is often associated with a milder long‐term phenotype, this case highlights that newborns compound heterozygous for p.Ser135Leu and a null variant are at risk of end‐stage liver disease if not immediately switched to a low‐galactose diet. Surprisingly, despite the transplant with an ostensibly normal liver and continued dietary galactose restriction, this patient continues to show mildly elevated RBC Gal‐1‐P and urine galactitol.

**Summary**

- Prolonged milk exposure resulted in life‐threatening liver disease in a newborn despite presence of a p.S135L GALT allele.
- Despite a liver transplant at age 2 months, both RBC Gal‐1‐P and urine galactitol remained mildly elevated through mid‐childhood.

## 1 Introduction

Classic Galactosemia is a rare inherited metabolic disorder caused by a deficiency in the galactose‐1‐phosphate uridylyltransferase (GALT) enzyme. Individuals with classic galactosemia exhibit severely reduced GALT activity, typically less than 1% in red blood cells [1]. In contrast, those with clinical variant galactosemia possess one or more hypomorphic *GALT* variants resulting in residual enzyme activity ranging from 1% to 10%, at least in some tissues [2]. The *GALT* p.Ser135Leu (c.404C>T) variant is strongly linked to clinical variant galactosemia [3].

Both classic and clinical variant galactosemia may present in newborns with failure to thrive, cataracts, anemia, and liver dysfunction unless a galactose‐restricted diet is implemented in the first days to weeks of life. While the residual GALT activity conferred by the S135L variant is thought to be partially protective in terms of long‐term outcomes [3], we present the case of a now 12‐year‐old male compound heterozygous for a novel null variant and S135L, who was untreated and presented in liver failure at age 2 months, requiring liver transplant. We review his pre‐ and post‐transplant biochemistry and developmental outcome.

## 2 Case

**Neonatal period**: The patient was born at 35 weeks, 6 days to a 39‐year‐old G1P1 mother and spent 4 days in NICU for hypoglycemia and hyperbilirubinemia treated with phototherapy. The initial newborn screening (NBS) sample, collected at approximately 30 h of life, revealed a GALT of 18.7 μM (*N* > 40.0), total galactose of 39.8 mg/dL (*N* < 15), and galactose‐only of 3.6 mg/dL, suggestive of GALT deficiency with an elevated calculated Gal‐1‐P of 36.2 mg/dL but complicated by a relatively high GALT activity level.

In addition to GALT and total galactose screening, NBS for galactosemia included testing for six common *GALT* variants. A single heterozygous variant, p.Ser135Leu (c.404C>T), was identified, resulting in a misinterpretation of the NBS report that the patient was a *GALT* variant carrier and not affected.

A second NBS sample, collected at age 7 days, was similarly concerning with a total galactose level of 35.3 mg/dL (*N* < 15) and galactose‐only of 3.5 mg/dL despite a surprisingly high GALT activity of 20.7 μM (*N* > 40.0). The infant was then referred to an NBS follow‐up center but was continued on breast milk and milk‐based infant formula with no call for urgent testing or follow‐up. At age 7 weeks, the baby presented to a local emergency department with decreased feeds, lethargy, and concern for an incarcerated hernia. A follow‐up abdominal ultrasound revealed diffuse ascites and a liver of normal homogeneous appearance without evidence of biliary dilatation. The baby was discharged home but presented again 1 week later with liver failure, hypoglycemia, disseminated intravascular coagulation, and encephalopathy. PT was 42.4 s (Normal 11.7–14.5), PTT 56.6 (22.9–33.9), and INR 4.7 (0.8–1.2). The baby was given transfusions of packed red blood cells, platelets, and fresh frozen plasma. Total bilirubin was 14.1 mg/dL (0.2–1.3), direct bilirubin 8.6 mg/dL (0.0–0.3), Alkaline phosphatase 1270 U/L (38–126), AST 325 U/L (3–34), and ALT 1091 U/L (15–41). Infectious work‐up was negative. Brain CT showed diffuse white matter edema.

**Liver transplant**: The baby was transferred to a tertiary care facility for further work‐up and management, where he showed little improvement while receiving galactose‐free nutrition. He ultimately received an orthotopic liver transplant at age 68 days, as his existing liver injury was considered irrecoverable. Consistent with this determination, examination of the explant revealed end‐stage liver disease with cirrhosis and fibrosis featuring bile duct proliferation, extensive clear cell changes of the liver cells, and extensive and severe cholestasis. The baby experienced a complicated post‐transplantation course, in particular punctuated by bile duct strictures that failed stenting and required a surgical hepatico‐jejunostomy revision.

**GALT activity and genetic testing**: Confirmatory red blood cell GALT activity, only available following the transplant, was reported as 15.9 U/g Hb from one sample and 14.6 U/g Hb (Normal ≥ 18.5 U/g Hgb) from a second sample collected 8 days later. These levels are high and seem inconsistent with a diagnosis of classic or clinical variant galactosemia; however, the patient received blood transfusions, ostensibly from a GALT+ donor, only days before each diagnostic sample was collected, meaning both samples were heavily contaminated with donor cells.

Follow‐up *GALT* sequencing revealed that, in addition to the known S135L *GALT* variant previously identified as part of the newborn screen, a novel out‐of‐frame small deletion (c.576_589delCAGTTTCCTGCCAG) was also present, resulting in a downstream premature stop codon, presumably in *trans* with the S135L. Additional pre‐transplant diagnostic work‐up including alpha‐1‐antitrypsin, serum copper, plasma amino acids, urine organic acids, and acylcarnitine profile were all non‐diagnostic for other causes of liver failure. No sequence variants were identified in *DGUOK* (deoxyguanosine kinase) exons or intron/exon boundaries, or in mitochondrial DNA. While whole exome sequencing to search for other possible genetic causes of the liver failure might be considered today, in 2012 when this patient was born, clinical access to whole exome sequencing was much more limited.

The patient continued to experience multiple medical complications, including eosinophilic esophagitis, which affected his eating. He wears corrective lenses for myopia but has no cataracts or other ophthalmologic abnormalities. His brain MRI at age 20 months showed mild diffuse cerebral volume loss but normal myelination.

**Developmental outcomes**: Developmental evaluation between ages 12 months and 2 years 8 months demonstrated a range of delays. Specifically, at 12 months, the patient's skills fell in the 8–10‐month range, and at age 16 months he was found to have functional skills scattered up to a 12‐month level, with more significant delays in language including limited vocal expression and language comprehension. At 23 months, the patient's overall skills were reported at the 12–15‐month level. At 2 years 8 months, he was found to be at the 18–24‐month developmental level, demonstrating difficulties with short attention, high activity, and self‐directed behavior.

However, later neuropsychological testing completed at ages 6, 9, and 11 years demonstrated age‐appropriate academic skills with strengths in verbal skills, learning and memory, and processing speed. Overall intellectual functioning was in the average range and appeared stable over time with full scale IQ (FSIQ) scores of 98, 101, and 102 reported at ages 6 years, 9 years, and 11 years 8 months, respectively. Some mild weaknesses were noted in visual–spatial and visual reasoning skills, and aspects of executive functioning (e.g., planning/organization, working memory), but concerns for attention reported earlier in childhood were no longer seen.

**Metabolites**: Following his liver transplant, the patient was continued on a galactose‐restricted diet and referred to our center for metabolic follow‐up. Despite a galactose‐restricted diet, his RBC Gal‐1‐P levels and urine galactitol have remained elevated, ranging from 1.7–2.8 mg Gal‐1‐P/100 mL red cells (*N* < 1) and 248–331 mmol galactitol/mol urine creatinine (N 2–36). Specifically, at age 4 years, RBC Gal‐1‐P was 1.2 mg/100 mL red cells (*N* < 1) and urine galactitol was 224 mmol/mol creatinine (N 2–36). At age 9 years, RBC Gal‐1‐P was 1.3 mg/100 mL red cells (*N* < 1). Additionally, commercial analysis of transferrin and apolipoprotein CIII isoforms and serum N‐linked oligosaccharides analyzed by matrix‐assisted laser desorption/ionization time‐of‐flight mass spectrometry were all reported as normal.

A galactose challenge was conducted at 9 months by giving the patient 50% soy formula and 50% galactose‐containing formula (2.3 g/kg/d galactose) over a 2‐week period. RBC Gal‐1‐P and urine galactitol levels post challenge were 2.3 mg Gal‐1‐P/100 mL red cells (N 0–1) and 286 mmol galactitol/mol urine creatinine (N 2–36). Although these levels were not substantially higher than those seen before the challenge, because they were above the normal range, the patient was returned to a galactose‐restricted diet, which has been maintained.

## 3 Discussion

This case teaches two important lessons. First, it is a cautionary tale illustrating that patients with clinical variant galactosemia, like patients with classic galactosemia, require immediate dietary restriction of galactose in the newborn period. An out‐of‐range newborn screening result for galactosemia should trigger immediate dietary intervention that can later be relaxed if follow‐up diagnostic testing deems it unnecessary. Patients with clinical variant galactosemia may experience milder long‐term outcomes than their counterparts with classic galactosemia, but that does not mean they also experience milder acute outcomes [3]. The acute symptoms experienced by the patient described here speak for themselves.

The second lesson of this case is that when total galactose metabolites are elevated, even if GALT activity is reported as relatively high, dietary intervention should not be delayed, especially if the baby shows early clinical signs consistent with classic galactosemia. Further, panel testing for common *GALT* variants is conclusive only when two pathogenic variants are identified and confirmed to be in *trans* (1 from each parent). If one or no recognized pathogenic variants are identified in a patient with elevated galactose metabolites, the presence of unidentified pathogenic variants must be considered. In such a case, full *GALT* gene sequencing is required to identify rare variants if they are present.

The relatively high RBC GALT activity reported for this patient both by newborn screening and following his liver transplant (15.9 and 14.6 U/g Hb with Normal ≥ 18.5 U/g Hb) seems at odds with his *GALT* genotype. We cannot explain the newborn screening result but believe the elevated diagnostic testing result is likely an artifact due to blood transfusions received only days before each diagnostic testing sample was collected. This is another important aspect of the cautionary tale—RBC enzymes measured shortly after a blood transfusion are likely confounded by the presence of donor cells in the sample. Of course, genetic testing may still be accurate if the sample is collected from another source, such as a buccal smear.

The *GALT* variant S135L is common among galactosemia patients of African, African‐American, Portuguese, and Brazilian ancestry and is associated with cryptic residual GALT activity, at least in some tissues [2, 4]. It is one of the most prevalent *GALT* alleles associated with clinical variant galactosemia [3]. Unfortunately, some infants homozygous for this variant have been missed by NBS [5], putting those infants at increased risk for acute disease. Indeed, a false‐negative NBS result may further delay intervention by causing healthcare providers to discount galactosemia in a differential diagnosis of symptoms [3, 5].

While it is tempting to draw conclusions from the metabolite levels reported here—specifically that comparable RBC Gal‐1‐P and urinary galactitol levels were seen both on dietary galactose restriction and following a 2‐week galactose challenge—this is speculation. However, that much higher RBC Gal‐1‐P levels were seen twice following galactose challenge in a different patient with S135L GALT who did not have a liver transplant [3] raises the intriguing possibility that GALT activity in the transplanted liver may have prevented a similar metabolite spike in the patient described here. That the RBC Gal‐1‐P and urine galactitol levels were not fully normalized in this patient following liver transplant may indicate that continuing GALT deficiency in all tissues other than liver, coupled with endogenous biosynthesis of galactose in those tissues, resulted in the accumulation of metabolites that, unlike plasma galactose derived from diet, were either not readily accessible to the liver (RBC Gal‐1‐P) or not readily metabolized by the liver (galactitol).

Finally, it is tempting to connect the current patient's encouraging developmental outcome in mid‐childhood with the GALT activity in his transplanted liver. However, the presence of his S135L allele suggests he may have had a mild long‐term outcome even without a liver transplant. It will be important to compare the metabolic and phenotypic outcomes of this patient with those of other galactosemia patients who have also experienced liver transplant to determine the true impact of GALT activity restricted to the liver.

## Author Contributions

Kara Simpson and Nicholas Ah Mew acquired data and drafted this case report. Erin L. MacLeod, Julia Clayton, and Nada A. Yazigi, each provided patient data and medical expertise. M. Estela Rubio‐Gozalbo, and Gerard T. Berry each provided additional expert analysis and interpretation of data. Judith L. Fridovich‐Keil assisted with the interpretation of data and drafted and edited portions of the manuscript. All authors have provided draft revisions and have approved the final version to be submitted.

## Ethics Statement

As per Children's National IRB guidance, case reports “do not meet the definition of research in either 45 CFR 46.102(l) or 21 CFR 56.102(e) and, therefore, do not require approval by the Children's National Institutional Review Board (IRB) as research.”

## Consent

The patient's parents have provided consent on behalf of their child.

## Conflicts of Interest

The authors declare no conflicts of interest.

## Data Availability

Anonymized data presented in this manuscript are available from the corresponding author, N.A., upon reasonable request.
